# Earnings management in state-owned enterprises: bringing publicness back in

**DOI:** 10.1007/s10997-021-09589-3

**Published:** 2021-07-01

**Authors:** Pasquale Ruggiero, Daniela Sorrentino, Riccardo Mussari

**Affiliations:** 1 Department of Business and Law, University of Siena, Siena, Italy; 2 Brighton Business School, University of Brighton, Brighton, UK; 3 Department of Economics Marco Biagi, University of Modena and Reggio Emilia, Modena, Italy

**Keywords:** Earnings management, Dimensional publicness, Financial accountability, State-owned enterprises

## Abstract

Financial accountability is a major issue for State-owned Enterprises (SOEs) especially because of the large amount of public resources invested in them and the social relevance of their performance. In the awareness that the public interest is increasingly pursued in abstract arenas, the investigation of accounting should be anchored to conceptual rather than contextual spaces. Building on the dimensional concept of publicness, this paper investigates the impact of three publicness dimensions (ownership, political control, and goal ambiguity) on earnings management (EM) in SOEs, a managerial practice that affects the quality of financial accountability. Drawing on data from a sample of 1200 Italian SOEs, the conditional revenue model (Stubben, 2010) is used to estimate their EM during the period 2009–2017. These EM estimates are then regressed against dimensions of publicness. Findings show that publicness is either neutral or relevant for the quality of SOEs’ financial accountability, depending on the dimensions analysed: while ownership and financial control are positively related to EM in SOEs, administrative control and goal ambiguity are not statistically significant predictors of EM. Moreover, the interaction of publicness dimensions does not affect EM in SOEs. Therefore, this paper shows that SOEs’ publicness is either irrelevant or detrimental to the quality of SOEs’ financial accountability, depending on the dimension of publicness considered. Efforts should be made to define policies and governance arrangements able to influence managers’ behaviour in a way that preserves SOEs’ financial accountability.

## Introduction

State-owned enterprises (SOEs) is the term most commonly used—in both the academic and practical spheres—to refer to enterprises ruled by private law in which state, regional and/or city governments have a significant role and control by dint of holding full, majority or significant minority ownership (Bel & Gradus, 2018; Garde-Sánchez et al., 2017). SOEs have been widely adopted in Europe—especially continental Europe—and are frequently engaged in the provision of public services of general economic and social interest, the performance of which is of great importance for communities (Cuervo-Cazurra et al., 2014; De Magalhães, 2010). The number and size of SOEs make them important players in the economy at the local, national, and international levels (Kowalski et al., 2013; OECD, 2015).

Due to all these aspects, the extent to which their financial reports can deliver accountability is a matter of great concern (Royo et al., 2019; Shaoul et al., 2012). Despite the growing concern over organisations’ non-financial performance (Kaur & Lodhia, 2019), financial accounting and accountability hold a key role not only in supporting governments and private investors’ decision-making, but also for legitimising policies of investment in/disinvestment from SOEs, especially in times of crises and austerity (Bracci et al., 2015; Lapsley et al., 2015). SOEs’ financial reports are the most important means of rendering financial accountability for public services, so any manipulation of the reported financial information would undermine SOEs’ answerability in this regard.

This article investigates earnings management (EM) as a phenomenon affecting the quality of SOEs’ financial accountability. EM entails the active intervention of managers in the process of financial performance representation, and thus undermines the quality of financial reporting to some degree (Ball, 2009; Ronen & Yaari, 2008). The EM phenomenon has been extensively studied in private contexts (Healy & Wahlen, 1999; Stolowy & Breton, 2004). However, the literature on EM in SOEs is relatively recent and limited, with studies exploring both private and public sector contextual variables. A predominance of studies focuses on the ownership structure as an explanatory variable of such accounting behaviour (Capalbo et al., 2018). Furthermore, empirical research has often focused on country-specific features or exceptional events in the life of an SOE (Lin et al., 2015; Liu & Lu, 2007; Wang & Yung, 2011). Not least, the relationship between EM and political elections has been also investigated (Capalbo et al., 2020; Chen et al., 2008; Cohen et al., 2019; Repetto, 2015).

Most of the literature seems to have neglected some specific features of the public domain to which SOEs belong, which are related essentially to the public interest they directly or indirectly pursue even if operating as private law-ruled organisations. In the awareness that the public interest is attained in increasingly abstract arenas, public sector accounting scholars are beginning to exit their niche and to engage in interdisciplinary research (Bruns et al., 2020; Jacobs, 2016), to the benefit of more generalisable understandings of public sector accounting and its social relevance. In this regard, Steccolini (2019) suggests shifting the focus from the public sector as a context of research—as opposed to the private one—to the publicness concept to pursue such an ambitious goal. Indeed, the publicness concept—pioneered by Bozeman (1987)—emerges as one capable of driving the attention towards the political authority exerted by public administrations over any organisation to pursue the public interest. Accordingly, this paper builds on the dimensional conceptualisation of publicness (Boyne, 2002; Bozeman, 1987; Rainey & Bozeman, 2000) and aims at exploring the relationship between EM and three publicness dimensions—ownership, control, and goal ambiguity—in SOEs. To this end, the paper draws on data from a sample of 1200 Italian SOEs and makes use of the conditional revenue model (Stubben, 2010) to estimate their EM during the period 2009–2017. These EM estimates are then regressed against publicness dimensions to analyse their relationships.

This paper offers both theoretical and practical contributions. First, it addresses the need to delve into the still little-explored world of SOEs, where the multiplicity of players and the increasing demand for accountability generates complex interrelations between accounting and accountability, calling for closer consideration (De Magalhães, 2010; Grossi et al., 2015). Therefore, extending the exploration of EM in SOEs to publicness dimensions contributes toward illuminating and explaining the impact of political authority on the quality of SOEs’ accountability for the use of public resources. In particular, this work is a valuable contribution to the accounting literature on EM in SOEs, which has so far mostly neglected the explanatory potential of concepts related to the pursuing of the public interest, such as publicness (Bruns et al., 2020; Steccolini, 2019). In bringing publicness back into accounting research and building on interdisciplinary discussions for the investigation of accounting phenomena, a further theoretical contribution stems from this work’s positioning at the conjunction of accounting and public administration literature. In particular, the dimensional approach to publicness allows for understanding the effects of the various dimensions through which political authority is exerted on the quality of SOEs’ financial accountability and in turn on citizens, politicians and managers’ decisions. Finally, the chosen empirical setting—besides being relevant for this study—is one in which EM in SOEs has not yet been studied (Capalbo et al., 2014). From a practical point of view, this paper offers insights on the impact that political authority—here conceptualised through dimensional publicness—has on EM in SOEs, offering cues for policy recommendations and suggestions for managing SOEs’ accountability relationships.

The paper proceeds as follows: the next section provides an outline of the financial accountability issue in SOEs and reviews the extant literature on EM in SOEs; the third section presents the theoretical conceptualisation of dimensional publicness, facilitating the development of hypotheses on the relationship between publicness and EM in SOEs; the fourth section lays out the research design, and the fifth reports the results of the analysis. Finally, the last two sections discuss the findings and draw some conclusions.

## EM and financial accountability in SOEs: a literature review

The notion of accountability can be summarised as a social relationship involving an *accountor*, who recognises an obligation to explain and justify her/his conduct to an *accountee* (Lindberg, 2013; Roberts & Scapens, 1985). As a social relationship, accountability can take many forms, both in terms of the subjects that it involves and the purposes it is deemed to serve (Bovens, 2007). Processes of externalisation and privatisation in the provision of public services have led to a broadening of both the subjects involved and the purposes for which accountability relationships arise (Godwin et al., 2019; Grossi et al., 2015). Because of these processes, financial accountability has gained primary relevance for SOEs. They provide services of public interest under the political influence of organisations at various governmental levels (Allini et al., 2016; Grossi & Thomasson, 2015), so there is great emphasis on how they spend public resources (Shaoul et al., 2012). As financial accountability regards how resources are allocated and recorded (Behn, 2001), accounting provides the mechanism that allows accountability for finance to be fulfilled.

The accounting literature posits that the preparation of accounting data implies the adoption of some degree of discretion, since it is often the result of estimates and conjectures elaborated by human beings (Watts & Zimmerman, 1978). Accordingly, managers are likely to maximise their own interests—that is, they tend to exercise that discretion to ‘manage’ accounting data and disclose financial and economic results to meet additional needs besides their truthful representation. The term EM refers to the active intervention of managers in the process of drafting organisations’ financial and economic performance data, which undermines the quality of their reporting to some extent (Ball, 2009; Ronen & Yaari, 2008). Consequently, this paper acknowledges EM as the manipulation of accounting information that affects the quality of SOEs’ financial accountability. The literature on EM in SOEs is relatively recent and largely unexplored (Capalbo et al., 2018), mainly due to the scant availability of data needed to estimate EM through well-recognised methodologies (Healy & Wahlen, 1999). To date, scholars have paid particular attention to the relationship between EM and the ownership structure (Capalbo et al., 2018), studied both in terms of ownership concentration (Bowen et al., 2008; Shleifer & Vishny, 1989; Warfield et al., 1995) and by looking at ownership features, such as the family nature of ownership (Cascino et al., 2010; Jiraporn & DaDalt, 2009) and the presence of institutional investors (Lemma et al., 2018). More recently, some scholars have gone beyond the ownership focus to study the relationship between EM and either the sustainability reporting disclosure (Carey et al., 2017; Liu & Lee, 2019) or the impact of the introduction of managerial performance appraisal systems (He et al., 2020) in SOEs. Furthermore, a relevant stream of literature has focused on how earnings are managed during exceptional business circumstances, such as IPOs (Cheng et al., 2015; Huang & Li, 2016) and privatisations (Chen et al., 2014), and on specific contexts, such as the Chinese milieu (Lei & Wang, 2019; Zheng et al., 2019). Notably, the peculiarities of the empirical contexts and the exceptionality of the events in which EM has been investigated provide contrasting results on the explanatory power of the variables examined. Additionally, results are likely to depend largely on the EM estimation methods adopted (Healy & Wahlen, 1999). For instance, Wang and Yung (2011) use discretionary accruals to estimate EM (Jones, 1991) and detect a negative relationship between public ownership and EM in SOEs. Nevertheless, these results also reflect the role played by the Chinese government, which has discouraged opportunistic behaviours on the part of SOEs and protected them in such a way as to minimise their incentives for EM. On the contrary, Guo and Ma (2015) use the Dechow and Dichev (2002) model on a sample of 1176 Chinese listed firms and detect a negative relationship between the number of shareholders and EM, possibly justified by the variance of interests when ownership is parcelled out among several shareholders.

Furthermore, some scholars have explored EM during political election periods. Electors have been found to be sensitive to SOEs’ financial performance—as a proxy of SOE managers’ good performance (Chen et al., 2008)—to the extent that they may proactively research SOE financial statements in runups to elections (Repetto, 2015). Therefore, politicians are likely to put SOE managers under pressure to disclose financial information functional to their own elections (Cohen et al., 2019). SOE managers themselves may also be prone to manage SOEs’ earnings to cater to politicians and safeguard their own appointments after elections (Capalbo et al., 2020).

The extant literature has investigated EM determinants in SOEs by exploring whether and how private or public sector contextual variables affect such accounting behaviour. As governance arrangements ruled by private law and providing public services of general and economic interest, SOEs are affected by varying degrees of political authority. SOEs are the result of the privatisation processes implemented while public ownership is totally or partially retained in some organisations, which are consequentially subject to a corresponding level of political influence. Privatising an organisation that has not been definitively hollowed out ensures that some control is maintained over publicly relevant outputs, while allowing its managers’ decision-making autonomy, to which a congruent degree of responsibility is connected (Koppell, 2006). SOEs are a type of governance arrangement in which private and public-like features coexist to provide public services. This suggests that it would be unsuitable to narrow the understanding of SOEs’ accounting and reporting behaviour to the effect of contextual variables—either private or public sector ones (Argento et al., 2019). On the one hand, the accounting literature could pave the way for further research aimed at producing more generalisable findings on EM in SOEs (Capalbo et al., 2014) and their capacity to pursue the public interest. On the other hand, public sector accounting scholars should acknowledge that the public interest is nowadays attained in more abstract arenas, so that the understanding of accounting phenomena in those arenas may be greatly enhanced by focusing on conceptual rather than contextual variables (Steccolini, 2019). In the attempt to enhance the knowledge on EM in SOEs, this paper welcomes the highlighted calls by using the publicness concept to search for explanations to EM in SOEs. Precisely, the dimensional publicness (Bozeman, 1987) provides a theoretical perspective that can support such a research trajectory. Publicness conceives organisations as affected by varying degrees of political and economic authority; thus, it promotes an understanding of organisations’ behaviour that transcends the contextual borders of both private and public sectors.

## Publicness in SOEs: a theoretical framework and hypotheses building

This section reviews the theoretical conceptualisation of publicness as it has been developed in the literature, and explains the version adopted in this paper. The concept of publicness is then applied to SOEs, explaining its relevance in those organisations. Finally, theoretical arguments are used to develop hypotheses about the publicness relationship with EM.

### The conceptual definition of publicness

The distinction between and the underlying criteria used to classify organisations as either private or public has long been a matter of great concern for the development of a specific management theory (Dahl & Lindblom, 1953; Nutt & Backoff, 1993). This distinction is considered fundamental for identifying the two types of organisations and associating specific organisational typologies, theories, and managerial models (Rainey & Bozeman, 2000).

To this end, three main classification approaches have been proposed: the core approach, the dimensional approach, and the environmental approach (Scott & Falcone, 1998). The core approach places a strong emphasis on the nature of the owners, classifying organisations according to the type of owner (Perry & Rainey, 1988). To be considered public, an organisation must be owned collectively by members of a political community that finance and strictly control its activities, while private organisations are owned by entrepreneurs or shareholders that mainly operate with the market logic. Despite its simplicity, the core approach has been strongly criticised, especially following recent public sector reforms, and is considered somewhat misleading in depicting real-life instances (Bozeman & Bretschneider, 1994). Indeed, publicly owned organisations may also operate by pursuing private-like objectives, and privately-owned organisations may operate in the public sphere by providing public services (Haque, 1996).

This evidence led to the abandonment of the core approach and a shift towards the dimensional publicness approach (Bozeman, 1987; Moulton, 2009), in which the level of publicness of an organisation depends on the relative degree of influence exercised on it by a political authority as opposed to an economic authority. An organisation is public as long as it is primarily constrained or enabled by a political authority, whereas economic authorities constrain and enable private organisations. The cornerstone of the dimensional approach is the assumption that the publicness of a given organisation does not stem from a single discrete attribute (as in the core approach). Rather, publicness is connected to the level of manifestation of some specific features that determine the degree and type of authority to which an organisation is subjected (Bozeman, 1987; De Magalhães, 2010). Some mix of political and economic authority, which determines its relative degree of publicness, influences every organisation. Therefore, the level of publicness of an organisation affects how it is managed, i.e., the way its managers behave and make decisions (Coursey & Bozeman, 1990).

The major implications of the dimensional approach lie not only in rejecting the idea of a wholly private or public organisation, but also in recognising that a given organisation may be more public in some regards and more private in others. The title of the well-known book by Bozeman (1987), *All organizations are public*, is emblematic in this respect. The dimensions traditionally recognised in the literature and used to define an organisation’s level of publicness are ownership, control, funding, and goal ambiguity (Boyne, 2002; Bozeman, 1987). The publicness/privateness of an organisation is defined according to the intensity of political/economic authority exerted—directly or indirectly—by either politics or the market on its management, authority exerted through ownership, control, funding, and goal ambiguity (Boyne, 2002; Rainey, 2009).

The conceptualisation of publicness has been further elaborated going beyond individual organisations and encompassing the environment in which they operate (Moulton & Bozeman, 2011). Considering that organisational outcomes can be affected by individual, organisational, and environmental factors operating at different levels (Heinrich & Lynn, 2002), the environmental publicness approach entails the incorporation of indirect influences of publicness on organisations. Hence, the assessment of publicness can extend to an organisation’s policy environment, and related measures such as the collective organisational publicness of organisations interacting in a policy environment or the public priority of a policy issue can be used (Miller & Moulton, 2013).

As this paper investigates an accounting practice—EM—carried out within organisations and does not focus on the specifics of a given policy area, the dimensional publicness conceptualisation is acknowledged to be the most appropriate for our purposes. Thus, in keeping with Bozeman’s (1987) concept of publicness, this paper focuses on understanding whether and how the existence of a greater or lesser degree of political authority exerted on SOEs could affect the quality of their financial accountability. Thus, this paper focuses on publicness dimensions as explanatory variables of the managerial behaviour (i.e., EM) that affects the quality of SOEs’ financial accountability. This allows achieving the sought shift from contexts to a conceptual space where explanations for the accounting and reporting behaviour of SOEs transcends the borders of both private and public sectors.

### Linking publicness and EM in SOEs: developing hypotheses

Building on the dimensional conceptualisation of publicness requires that SOEs—by virtue of the political authority maintained—be distinguished by varying levels of publicness dimensions. The literature identifies four dimensions that constitute the concept of publicness: ownership, control, goal ambiguity, and funding. Depending on the institutional environment within which these dimensions exist, they define an SOE’s publicness, but each of them may affect EM differently. In this paper, however, due to the institutional environment of the SOEs analysed, not all dimensions will be considered for the development of hypotheses. Funding refers to the proportion of an organisation’s financial resources coming from governmental sources, as opposed to those supplied directly by the market (Bozeman & Crow, 1990). According to the dimensional approach to publicness, it can be argued that every organisation—regardless of its legal status—derives financial support from a wide range of sources, including profits from markets, profits from government contracts, government subsidies or direct government appropriations (Perry & Rainey, 1988). Thus, the greater the government endowments for an organisation are, the greater the level of political authority to which that organisation is subjected (Bozeman, 1987; Rainey & Bozeman, 2000). While this notion of funding coherently adapts to the assessment of publicness in organisations such as governmental agencies, it cannot be transferred to SOEs as such. Government appropriations to private organisations that operate in competitive markets are meticulously regulated—if not entirely forbidden—in many jurisdictions, as they alter the proper functioning of the market and undermine consumers’ rights (Fox & Healey, 2013). Considering this fact and the pertinence of this reasoning with our empirical setting, the funding dimension has not been included in the theoretical framework of this paper.^1^

#### Ownership

Ownership was used to distinguish the behaviour of private and public organisations long before the rise of the dimensional approach to publicness (Miller & Moulton, 2013). Adopting the dimensional approach entails considering public ownership as a continuous variable of publicness, and thus acknowledging that political authority exerted on a given organisation may vary as levels of public ownership vary. This dimension of publicness is of relevance for SOEs, where public ownership is purposefully maintained to ensure the provision of services of general economic and public interest (Calabrò et al., 2013). Higher levels of public ownership in SOEs thus correspond to a related public interest, that is safeguarding community access to those services.

Capalbo et al. (2018) highlight that there are as many reasons to expect a positive relationship between the ownership of SOEs by political communities and EM as there are reasons to presume a negative one. Arguments in favour of a positive relationship rely on: (a) the expectation of a relatively lower quality of corporate governance in SOEs, which is often linked to a greater degree of managerial discretion (Shleifer, 1998); (b) the greater heterogeneity of SOEs’ *accountees* (Grossi & Thomasson, 2015), which increases the potential addressees of SOEs’ reporting (Bruton et al., 2015) and creates incentives for EM; (c) the fact that SOEs’ economic and financial results impact a quantitatively and qualitatively unidentifiable group of subjects (the community acting as the residual owner), thus decreasing the expectation that reporting of those results will be monitored, as compared to the alternative hypothesis of readily-identifiable private investors (Jones, 1991); and (d) the limited technical expertise of the addressees of SOEs’ reporting (Koh, 2003). These arguments are further supported by the property rights theory, according to which residual claimants of public ownership—citizens, bureaucrats, and politicians—have fewer property rights than private owners (Peda et al., 2013), and public sector managers are thus less pressured to make effective decisions in the public interest rather than their own self-interest (Asher et al., 2005). There are, however, other perspectives supporting arguments in favour of a negative relationship between public ownership and EM. Firstly, the demand hypothesis (Kim & Yi, 2006) suggests that more numerous and heterogenous ‘eyes’ on SOE performance incentivise managers to increase the quality of reporting (Burgstahler & Dichev, 1997). Furthermore, SOE managers are often appointed on the grounds of political rather than business rationales (Cheng et al., 2015), and thus have no interest in practising EM (Fan et al., 2007). Finally, while tax minimisation is a recurrent explanation for EM in private organisations (Lin et al., 2014), it is a less likely one in SOEs, where the owner and the tax recipient may be the same.

The literature has by no means reduced uncertainty regarding the relationship between EM and ownership; there are studies which provide contrasting results. Some support the existence of a positive relationship between ownership and EM (Chen et al., 2014), while others suggest a negative relationship between them (Aharony et al., 2010; Wang & Yung, 2011). As explained above, these contrasting results often stem from the varied EM estimation models used, and/or from contextual peculiarities and the exceptionality of events during which empirical observations have been made. Therefore, while the statistical significance of previous studies suggests the presence of some sort of relationship between public ownership and EM, the opposing theoretical arguments and the contrasting empirical results of these studies do not definitively establish the direction of said relationship. Hence, the hypotheses regarding ownership can be formulated as follows:

##### H1a

The higher the level of public ownership in SOEs, the more EM is practised by their managers.

##### H1b

The higher the level of public ownership in SOEs, the less EM is practised by their managers.

#### Control

Control refers to the level of influence exerted directly or indirectly on organisations by political subjects (e.g. authorities) as opposed to economic forces (Andrews et al., 2011; Bozeman, 2013; Moulton, 2009). Regardless of the type of ownership, an organisation may be more or less public in this respect depending on the degree of its compliance with governmental policies and regulations. Forms of political control—exerted by governmental ministries and regulatory bodies—refer to and involve various organisational activities, including audit, inspection, performance reporting, the submission of plans, limits on budgetary autonomy, and price regulations (Andrews et al., 2011; Ryan et al., 2019). Hence, this dimension of publicness entails some degree of subjection to either administrative or financial regulations, though these control mechanisms can also be combined (Ashworth et al., 2002) and could well affect an organisation’s financial performance. In SOEs, the control dimension of publicness is by nature relevant. According to our definition, although SOEs are organisations ruled by private law, they are characterised by varying degrees of influence exerted by political subjects, linked to the need to ensure that the public interest is served in their processes and outputs (Bruton et al., 2015).

The literature on publicness has traditionally associated administrative control with constrained managerial discretion, as it sets boundaries for the adoption and the scope of possible managerial actions (Berry, 2005; Nutt & Backoff, 1993). Andrews et al. (2011) also observe how organisations that are under greater political control—as opposed to economic control—are likely to be subject to multiple sources of authority. This suggests that increasing levels of administrative control in SOEs would not only limit managerial discretion in several respects, but also increase managers’ awareness that their processes and outputs—including financial and economic performance—will be validated by a multiplicity of controllers who hold the power to enforce sanctions if those processes and outputs do not comply with regulations. Yet, when considering financial control (such as price regulation), the regulatory capture concept developed in the economics literature provides opposing theoretical arguments. Regulatory capture refers to the process through which special interest groups can affect state intervention and is commonly used to explain the relationships between governments, regulators (when different from governments), and firms operating in regulated industries (Dal Bó, 2006). The crux of regulatory capture models is information asymmetry, the source of regulatory discretion that makes regulatory capture possible (Laffont & Tirole, 1993). Indeed, in regulated industries, firms hold private information on their costs and technologies. As regulatory agencies have the time and expertise to acquire that information—unlike governments—they are liable to be bribed by firms, which attempt to ‘capture’ government decision-making to safeguard their welfare. Additionally, in accounting literature, control mechanisms such as price regulations are considered to be costly regulations for firms which are likely to have an impact on their accounting choices (Watts & Zimmerman, 1978). In the end, because of the regulatory capture concept and the cost of regulations for firms, those operating in price-regulated industries could be more incentivised to exploit accounting discretion to maximise their interests.

The literature supports both theoretical expectations. On the one hand, decreasing levels of political control—i.e., the administrative control exerted through the monitoring of organisations’ compliance with formal rules and the enforcement of regulations—provide greater leeway for managers to manipulate financial information (Aharony et al., 2010). On the other hand, when subject to price regulations, organisations are incentivised to exploit the information asymmetry characteristic of regulated industries to manage earnings, to increase the likelihood of approval of price increase requests (Bowman & Navissi, 2003; Lim & Matolcsy, 1999). Based on these different expectations regarding the impact of administrative and financial control mechanisms on EM, two different hypotheses can be formulated:

##### H2a

The higher the level of administrative control in SOEs, the less EM is practised by their managers.

##### H2b

The higher the level of financial control in SOEs, the more EM is practised by their managers*.*

#### Goal ambiguity

This latter dimension of publicness refers to some peculiar features of public organisations’ goals, regarding their organisational focus or agenda (Goldstein & Naor, 2005) due to the multidimensionality of the public interest pursued by a political subject through exerting its authority. Both theoretical and empirical contributions on this topic have emphasised that public organisations have distinctive, multiple, and vaguer goals than their private counterparts (Bozeman & Bretschneider, 1994; Rainey et al., 1995). To achieve the collective purpose of their actions, public organisations pursue goals regarding aspects such as equity, accountability, fairness, and justice (Van der Walle et al., 2008). And the fact that public organisations’ goals are imposed—directly or indirectly—through the political process rather than selected by managers tends to make them even vaguer (Dahl & Lindblom, 1953). This set of circumstances is encapsulated in the concept of goal ambiguity, defined as ‘the extent to which an organisational goal or set of goals allows leeway for interpretation, when the organisational goal represents the desired future state of the organisation’ (Chun & Rainey, 2005, p. 2). Political interference from various constituencies, the existence of different interest groups and authorities, the typical trade-offs amid competing appeals, and the unavailability of clear and applicable indicators contribute to making organisational goals ambiguous (Rainey & Jung, 2015). This implies that there will tend to be a correspondence between an organisation’s publicness and higher levels of goal ambiguity.

The relevance of this dimension of publicness in SOEs is self-evident. As SOEs are accountable to a multiplicity of stakeholders, these organisations are induced to formulate goals that can respond simultaneously to varying and possibly contrasting needs, and that are both economic and socially relevant (Royo et al., 2019). Higher levels of goal ambiguity in SOEs reflect the attempt to encompass such divergent typologies of objectives, and the attempt to satisfy a multiplicity of accountability demanders.

The management control literature suggests that the multiplicity and vagueness of organisational goals increase managers’ discretion (Lipsky, 1980; Maynard-Moody & McClintock, 1987). Multiplicity, conflict, and vagueness are all attributes of goals that give managers the opportunity to select and interpret them in a way that determines change or stability within organisations. As Merchant and Van der Stede (2012) state, ‘specificity of expectations is one of the elements necessary for the implementation of tight result controls’ (p. 124). Goal ambiguity also undermines organisations’ accountability, as it reduces the possibility of developing appropriate and consistent performance evaluation criteria (Merchant & Van der Stede, 2012; Rainey & Jung, 2015). Financial performance is often used as a proxy for management’s contribution to fundamental organisational objectives, as direct measures of managerial performance in that respect are rarely possible (Kauhanen & Piekkola, 2006). Hence, higher levels of goal ambiguity in SOEs can be expected to lend their managers increasing discretion to practise EM. Managers may resort to multiple and even contrasting reasons to justify their conduct and the financial performance through which that conduct will be appraised. Thus, the third hypothesis is:

##### H3

The higher the SOE’s goal ambiguity, the more the EM is practised by its managers.

## Research design

The Italian context has been chosen here as the empirical setting to test the hypotheses on the relationship between publicness dimensions and EM in SOEs. Over the last few decades, Italian public sector reforms have evidenced the Italian Legislature’s will to introduce market-based principles into the system of public service provision, concurrently stressing the importance of applying a public-based perspective in the production and provision of specific goods and services (Cafferata, 2010; Monteduro, 2014). As compared to other countries, the tradition of State intervention in society—and the economy—has undoubtedly favoured the establishment of firms regulated by private law but totally or in large part owned by public administrations for the provision of public services of general economic interest (Pollitt & Bouckaert, 2017).

Consistent with the definition of SOEs provided in this paper, the sample used for the analysis comprises organisations owned by central and/or local (regional and municipal) governments with a share between 0.01 and 100 per cent.

The analysis entails two steps: (1) estimation of EM within the sample and (2) assessment of its relationship with the three publicness dimensions. Financial data was collected through the Bureau Van Dijk AIDA database. Non-financial information was collected manually for each variable, as explained later in this section. The AIDA database contains comprehensive information on about one million private and public Italian companies going back as far as 10 years. It includes, among other data, companies’ financial statements and indices, from which most of the required information was extracted for this study. Furthermore, AIDA enables the classification of companies by type of shareholder, and ‘State, governmental departments and local authorities’ is reported as a specific category of shareholder. Considering only limited companies not involved in insolvency procedures, 2414 companies owned by public administrations with a percentage ranging from 0.01 to 100 per cent were extracted.^2^ The time frame considered extends from 2009 to 2017, thus including the earliest and latest financial years for which information was available when the database AIDA was accessed (May 2019). The resulting sample comprises 1200 Italian SOEs with fully available data for which a data panel was built.

In the first step of the analysis, the amount of EM for each SOE in the sample was estimated using the ‘conditional revenue model’ developed by Stubben (2010). This model proxies EM with abnormal accounts receivable derived from the estimation of expected receivables, and measures EM through revenues rather than earnings. Other explanatory variables in the model are those linked to the receivables policy of a company: financial strength (proxied by size), stage in the business cycle (proxied by age), and operational performance (proxied by revenue growth rate and gross margin). For these characteristics, the Stubben model (2010) is considered less biased for measuring EM (Capalbo et al., 2014, 2018; McNichols & Stubben, 2018) than previously adopted models based on aggregate accruals (seeDechow & Dichev, 2002; Dechow et al., 1995; Jones, 1991). Equation (1) shows the Stubben model (2010) used in the first step of the analysis.

1 $$\Delta {\text{AR}}_{{{\text{it}}}} \; = {\text{ }}\alpha \; + {\text{ }}\beta {\text{1 }}\Delta {\text{R}}_{{{\text{it}}}} \; + {\text{ }}\beta {\text{2 }}\Delta {\text{R}}_{{{\text{it}}}} \; \times {\text{ SIZE}}_{{{\text{it}}}} \; + {\text{ }}\beta {\text{3 }}\Delta {\text{R}}_{{{\text{it}}}} \; \times {\text{ AGE}}_{{{\text{it}}}} \; + {\text{ }}\beta {\text{4 }}\Delta {\text{R}}_{{{\text{it}}}} \; \times \;{\text{AGESQ}}_{{{\text{it}}}} \; + {\text{ }}\beta {\text{5 }}\Delta {\text{R}}_{{{\text{it}}}} \; \times {\text{ GRRP}}_{{{\text{it}}}} + {\text{ }}\beta {\text{5 }}\Delta {\text{R}}_{{{\text{it}}}} \times {\text{ GRRN}}_{{{\text{it}}}} + {\text{ }}\beta {\text{6 }}\Delta {\text{R}}_{{{\text{it}}}} \times {\text{ GRM}}_{{{\text{it}}}} \; + {\text{ }}\beta {\text{7 }}\Delta {\text{R}}_{{{\text{it}}}} \; \times {\text{ GRMSQ}}_{{{\text{it}}}} \; + {\text{ }}\varepsilon _{{{\text{it}}}}$$

where Δ = annual change. AR_it_ = accounts receivables. R_it_ = total revenue. SIZE_it_ = logarithm of total assets.AGE_it_ = logarithm of the number of years since firms’ establishment.AGESQ_it_ = the square of the variable AGE.GRRP_it_ = industry median-adjusted change in revenues multiplied for a dummy variable equal to 1 if the GRR takes a positive value for firm *i* in year *t*, otherwise 0.GRRN_it_ = industry median-adjusted change in revenues multiplied by a dummy variable which is equal to 1 if the industry median adjusted change in revenues in year *t* for firm *i* is negative, otherwise 0.GRM_it_ = the industry median-adjusted gross margin.GRMSQ_it_ = the square of the variable GRM.

EM estimates obtained from the first step of the analysis were used in the second step to run a regression aimed at assessing the relationship between EM estimates and the three dimensions of publicness considered in the paper.

The method of operationalisation of publicness dimensions is described here below. As for public ownership, the use of the percentage of public ownership in the sampled SOEs was self-evident.

The control dimension of publicness was categorised as administrative and financial at the empirical level, leading to the use of two different measures to represent this dimension in the analysis. First, given that organisations providing public essential services are subject to a higher level of political authority as they have to comply with more stringent regulation of their activities, this aspect of control was operationalised by distinguishing public services as essential or non-essential.^3^ To this end, the sampled SOEs were classified according to the ATECO 2007 classification (see Table 6 in the Appendix).^4^ Public services considered essential by Italian legislation were listed, and each of them was coupled with the ATECO 2007 nomenclature coding. Because the AIDA database reports ATECO 2007 coding for each company, it was possible to identify SOEs in the sample that provide essential public services and are subject to stricter administrative control. Second, the existence of price regulations was used to differentiate SOEs subject to higher degrees of financial control. In the Italian context, price-regulated sectors are: (1) transportation, (2) communication, (3) energy and environment, and (4) financial and insurance services.^5^ The ATECO 2007 classification was used for this measure as well (see Table 6 in the Appendix).

To operationalise goal ambiguity, this study followed the lead of previous contributions on the topic in using mission comprehension ambiguity and the methodology for its calculation (Cochran & David, 1986; Rainey & Jung, 2015). Mission comprehension ambiguity refers to the degree of interpretative leeway an organisation’s mission allows in comprehending, explaining, and communicating organisational missions (Daft, 2004). Goal ambiguity has been calculated through the Gunning-Fog index (GFI), elaborated upon by Gunning and Kallan, (1994). By providing information on the readability of a mission statement, the GFI captures the average sentence length of the mission and its frequency of multisyllabic words—measuring its ‘fog’ in a 0–100 range of possible values, in which a higher score indicates higher mission ambiguity. The mission statements of the SOEs included in the sample were collected manually from SOEs’ websites and the official documents published thereon (such as Statutes and Annual Reports). When mission statements were not available on SOEs’ websites, the search was extended to public administrations’ websites, as they are mandatorily required to disclose information on the organisations that they own.^6^ GFI was calculated by summing the average sentence length of the mission statements and the percentage of hard words in the statements (hard words are those composed of 3 or more syllables) (Chun & Rainey, 2005).

To detect the strength and direction of the relationships among the three elected publicness dimensions and the EM estimates resulting from the first step of the analysis, a regression model was built as follows:

2 $$|{\text{DA}}_{{{\text{it}}}} |{\text{ }} = {\text{ }}\lambda _{0} + {\text{ }}\lambda _{{\text{1}}} {\text{PO}}_{{{\text{it}}}} + {\text{ }}\lambda _{{\text{2}}} {\text{ADCONTR}}_{{{\text{it}}}} + {\text{ }}\lambda _{{\text{3}}} {\text{FICONTR}}_{{{\text{it}}}} + {\text{ }}\lambda _{{\text{4}}} {\text{GA}}_{{{\text{it}}}} + {\text{ }}\lambda _{{\text{5}}} {\text{SIZE}}_{{{\text{it}}}} + {\text{ }}\lambda _{{\text{6}}} {\text{ROA}}_{{{\text{it}}}} + {\text{ }}\lambda _{{\text{7}}} {\text{LEV}}_{{{\text{it}}}} + {\text{ }}\lambda _{{\text{8}}} {\text{NOI}}_{{{\text{it}}}} + {\text{ }}\varepsilon _{{{\text{it}}}}$$

where |DA_it_| is the absolute value of the log of residuals of Eq. (1)^7^; PO_it_ represents the percentage of ownership in the SOEs by state, governmental departments, and local authorities; ADCONTR_it_ represents political control over administrative issues and is a dummy variable that equals 1 if the SOE provides a public essential service, otherwise 0; FICONTR_it_ stands for control over financial issues and is represented by a dummy variable equal to 1 if the SOEs operates in a price-regulated sector, otherwise 0; GA_it_ is goal ambiguity, measured by GFI. Some control variables were included in the regression model to account for SOE size (SIZE_it_)—defined as the SOEs’ total assets—and financial performance (leverage—LEV_it_, return on assets—ROA_it_, and non-operating income NOI_it_).

## Empirical results

### Descriptive statistics

Table 1 below shows the descriptive statistics for each variable in Eq. (2), for the whole sample of 1200 SOEs in the period 2010–2017.^8^

**Table 1:** Descriptive statistics

Stats	Variables					
	PO	GA	SIZE	ROA	LEV	NOI
Mean	85.10	27.36	3.76	3.29	8.77	170.04
p50	99.98	26.30	3.68	2.22	3.40	− 43.97
sd	21.98	6.50	.92	13.02	68.01	21,118.37
Min	0	11.47	1.00	− 732.10	− 951.46	− 697,404
Max	100	67.9	7.88	100.75	5304.61	943,623

The average level of public ownership—approximately 85 per cent—shows that Italian public administrations retain a considerable role in the provision of some services. This datum, coupled with the average size of SOEs—around 3.8 billion euro—highlights the economic and social relevance of SOEs in the context analysed. Considering the differences between organisations subjected to higher degrees of administrative control, it can be noted in any case that the level of public ownership is higher in organisations providing essential public services. In contrast, the average level of public ownership held in organisations operating in price-regulated sectors is slightly below the overall average. Since GFI can assume values from 0 to 100, and because the higher the index value, the higher the ambiguity of a mission statement, the recorded GFI mean value indicates a relatively strong level of mission comprehension clarity for SOEs for which mission statements were collected (approximately one-third of the sample). More precisely, GFI values range from 11.47 to 67.9, with an average value of approximately 27.

As for the financial performance of Italian SOEs, the profitability of their investments is almost stable across the years analysed, as shown by mean ROA (3.29) and ROA range (between 3.51 and 3.08). Regarding the use of external financial resources, LEV mean values range from a minimum of 5.9 to a maximum of 11.9, without showing a stable trend across the years analysed. Finally, the trend in NOI mean values for the years under analysis indicates a change in Italian SOEs’ non-operating activities in contributing to the production of value. Specifically, mean NOI went from 325.784 in 2010 to 1342.126 in 2017 (see Table 7 in Appendix).

Correlation analysis was also carried out (see Table 2). In this analysis, the dummy variables included in the regression model, i.e., ADCONTR and FICONTR, were considered. As shown in Table 2, all variables, except ADCONTR and FICONTR, have a low value of correlation with the other variables included in the regression model. The correlation value between ADCONTR and FICONTR is positive (0.9337). This is justified by the fact that the two variables belong to the same dimension of publicness, i.e., control, but operationalise different aspects. Therefore, the correlation values reported for all the variables denote a problem of multicollinearity between ADCONTR and FICONTR.

**Table 2:** Correlation analysis

	PO	ADCONTR	FICONTR	SIZE	ROA	LEV	NOI
PO	**1.0000**						
ADCONTR	**0.0215**	**1.0000**					
	0.0353						
FICONTR	**0.0297**	**0.9337**	**1.0000**				
	0.0036	0.0000					
SIZE	**0.0064**	**0.2618**	**0.3001**	**1.0000**			
	0.5292	0.0000	0.0000				
ROA	** − 0.0908**	**0.0047**	**0.0064**	** − 0.1064**	**1.0000**		
	0.0000	0.6439	0.5333	0.0000			
LEV	** − 0.0016**	** − 0.0100**	** − 0.0132**	** − 0.0024**	** − 0.0127**	**1.0000**	
	0.8774	0.3283	0.1974	0.8116	0.2148		
NOI	** − 0.0050**	** − 0.0038**	** − 0.0025**	**0.0625**	** − 0.0145**	** − 0.0019**	**1.0000**
	0.6230	0.7081	0.8088	0.0000	0.1545	0.8514	

Bold values are the correlation results. The other values are the corresponding p-values

As for the other variables, all correlation values are below 0.9, as suggested by Hair et al. (2014). This result is confirmed by the Variance Inflation Factor (VIF) (see Table 3) values. In fact, all VIF values are under 4 for all variables except ADCONTR and FICONTR, which means that there is a high tolerance value, denoting a small degree of multicollinearity, i.e., the other independent variables collectively have no substantial amount of shared variance (Hair et al., 2014). By contrast, the VIF values for ADCONTR and FICONTR (respectively 7.83 and 8.03) suggest a problem of multicollinearity between them. The following was attempted to solve this issue: first, the regression model has been run with both variables; then, given that ADCONTR was found not to be statistically significant related to |DA|, it was dropped from the model (see Table 8 in Appendix).

**Table 3:** Collinearity Diagnostics: VIF

Variable	VIF	SQRT VIF	Tolerance	R-Squared
PO	1.01	1.00	0.9903	0.0097
ADCONTR	7.83	2.80	0.1277	0.8723
FICONTR	8.03	2.83	0.1246	0.8754
SIZE	1.12	1.06	0.8916	0.1084
ROA	1.02	1.01	0.9784	0.0216
LEV	1.00	1.00	0.9996	0.0004
NOI	1.00	1.00	0.9955	0.0045

### Regression analyses

Adopting the Stubben (2010) ‘conditional revenue model’, Eq. (1) was run after deflating all revenue and accrual variables by SIZE. Each model input variable was winsorised at 5 per cent and heteroscedasticity was estimated, following Breusch-Pagan (1980) and Cook-Weisberg (1983) (see Table 9 in Appendix). Given that the variance of estimated errors was seen to vary widely, a biweight robust regression was conducted (Tukey, 1970). This regression proves suitable for cases of relevant heteroscedasticity: it first fits the regression calculating the Cook’s D, thus excluding any observation for which D > 1, then works iteratively, performing a regression, calculating case weights from absolute residuals, and regressing again, using those weights until the maximum change in weights drops below the tolerance level. Weights derive from two weight functions, Huber weights and biweights.

The absolute value of the residuals from the Stubben model (2010) provides an estimate of abnormal accounts receivable or discretionary accruals (labelled |DA_it_|), which is used as the proxy of EM in SOEs. Therefore, the absolute value of residuals resulting from Eq. (1) is used as the dependent variable in the second step of the analysis, assessing the relationship between EM and the publicness dimensions discussed above (public ownership, control, and goal ambiguity).

A further challenge in this analysis was the unavailability of SOEs’ mission statements in their official documents and websites. This situation made it difficult to gather the data needed to measure mission comprehension ambiguity and consequently to operationalise the GA variable. In the end, 573 mission statements out of 1,200 were collected. To overcome this problem, a different research strategy was adopted.^9^ Equation (2) was run on the whole sample both including and excluding GA (See Table 10 in Appendix). Results show that GA does not predict the dependent variable |DA| and does not affect the causal relationship between the latter and the other independent variables. Thus, the GA variable was deleted from Eq. (2).

Table 4 below depicts the distribution of parameter estimates for Eq. (2), after excluding GA.

**Table 4:** OLS-biweights regression analysis

VARIABLES	OLS-biweights
	|DA|
PO	0.00472***
	(0.000804)
FICONTR	0.418***
	(0.0370)
SIZE	− 0.539***
	(0.0203)
ROA	0.00206
	(0.00137)
LEV	0.00118***
	(0.000426)
NOI	2.80e-06***
	(8.35e-07)
Constant	− 4.269***
	(0.102)
Observations	9,598
R-squared	0.075
Fstat	130.4
Prob > F	0

Robust standard errors in parentheses

***p < 0.01; **p < 0.05; *p < 0.1

The results of all estimates were highly significant, indicating that the regression used is effective in estimating EM.

Within a 95 per cent confidence interval, all variables except ROA are shown to be in an extremely significant relationship to |DA|. PO and FICONTR publicness variables—two of the explanatory variables of the analysis—are highly predictive of |DA| in Italian SOEs. Public ownership is positively associated with |DA|, which leads to the rejection of the theoretical expectations underpinning H1b. On the contrary, results show that managers of SOEs with higher levels of public ownership are more likely to practise EM, thus confirming H1a. Financial control is positively associated with |DA|, implying that higher degrees of control over SOEs’ financial issues increase the likelihood that SOEs’ managers practise EM. Thus, the findings support H2b, which postulated that higher degrees of financial control by public authorities would incentivise opportunistic accounting behaviours.

All of the variables controlling for SOEs’ financial performance are shown to be significantly related to EM, except for ROA. SIZE is found to have a strongly negative association with EM, thus contrasting with extant literature, according to which EM is practised more in larger companies (Burgstahler & Dichev, 1997). LEV results support the idea that SOEs characterised by a higher level of external financing (as opposed to equity resources) are run by managers keener to practise EM. Finally, NOI is strongly and positively associated with |DA|, leading to a twofold consideration. On the one hand, a relatively good performance in the non-operating area of management may induce managers to use EM practices to mask a relatively poorer performance in the operating area. In this case, the manipulation of financial information would be aimed at balancing performance in the two areas. On the other hand, if both areas are performing increasingly well, managers may be interested in reporting financial results as constant over time and masking outlier performance. In such cases, the manipulation of financial information would be aimed at reducing the reported performance of both operating and non-operating areas.

### Further investigations

Further investigations were carried out to consider effects on EM stemming from the interaction among those dimensions, as well as time effects. These additional analyses were done separately and together (see Table 5), to check the persistency of the effects of the different variables included in the regression model. These interaction analyses were carried out in the awareness that though each publicness variable represents a different dimension of publicness—consistent with the theoretical framework adopted in this paper—they all pertain to the same phenomenon. The effects of publicness on EM stem from each dimension of publicness, however, those effects may be strengthened through interactions among said dimensions and converge to determine the level of publicness.

**Table 5:** Regression analysis with interaction effects among publicness variables and time effects

VARIABLES	OLS-biweights |DA|	OLS-biweights |DA|	OLS-biweights |DA|	OLS-biweights |DA|
PO	0.00472***	0.00362***	0.00468***	0.00353***
	(0.000804)	(0.00111)	(0.000801)	(0.00110)
FICONTR	0.418***	0.219	0.419***	0.211
	(0.0370)	(0.142)	(0.0368)	(0.141)
SIZE	− 0.539***	− 0.538***	− 0.539***	− 0.538***
	(0.0203)	(0.0203)	(0.0202)	(0.0202)
ROA	0.00206	0.00206	0.00213	0.00214
	(0.00137)	(0.00137)	(0.00136)	(0.00136)
LEV	0.00118***	0.00117***	0.00109**	0.00108**
	(0.000426)	(0.000427)	(0.000425)	(0.000425)
NOI	2.80e-06***	2.82e-06***	2.98e-06***	3.00e-06***
	(8.35e-07)	(8.35e-07)	(8.32e-07)	(8.32e-07)
FICONTR*PO		0.00233		0.00243
		(0.00160)		(0.00160)
2011			− 0.220***	− 0.221***
			(0.0701)	(0.0701)
2012			− 0.370***	− 0.370***
			(0.0701)	(0.0701)
2013			− 0.284***	− 0.284***
			(0.0701)	(0.0701)
2014			− 0.382***	− 0.383***
			(0.0701)	(0.0701)
2015			− 0.511***	− 0.512***
			(0.0701)	(0.0701)
2016			− 0.519***	− 0.519***
			(0.0702)	(0.0701)
2017			− 0.464***	− 0.464***
			(0.0701)	(0.0701)
Constant	− 4.269***	− 4.178***	− 3.919***	− 3.824***
	(0.102)	(0.120)	(0.112)	(0.128)
Observations	9,598	9,598	9,598	9,598
R-squared	0.075	0.076	0.084	0.084
Fstat	130.4	112.2	67.76	63.15
Prob > F	0	0	0	0
AIC	1.090	1.090	1.083	1.082
BIC	1.095	1.096	1.093	1.094

Robust standard errors in parentheses

***p < 0.01; **p < 0.05; *p < 0.1

Nevertheless, results from further investigations highlight that EM in SOEs is not influenced by the interaction between the publicness dimensions considered in Eq. (2), suggesting that the interaction among the publicness dimensions used in the regression model do not affect the decisions of SOEs’ managers in practising EM.

As shown in Table 5, findings regarding time effects underline the existence of exogenous variables that are significantly associated with EM. These results lead to two considerations. First, the exogenous variables are negatively related to EM in SOEs. Second, their relationship with EM increased over the years analysed. The four models used for the analysis report a steadily increasing R-squared value. This slight increase in the R-squared value may have two explanations. First, the results of the regression are based on a large sample composed of subjects belonging to very different economic sectors. This implies that all regressors could exert their effects differently on SOEs’ managers and their accounting behaviours, due to the different pressures on financial accountability. Second, observations regard a very long period, the main feature of which was an economic crisis that required different types of intervention by political authorities depending on the economic and social relevance of the various economic sectors to which the SOEs analysed belong. In any case, the R-squared value trend does not diminish the reliability of the relationships among the publicness dimensions analysed and EM in SOEs.

Additionally, the Akaike information criterion (AIC) (Akaike, 1974) and the Bayesian information criterion (BIC) (Stone, 1979) were computed to measure the level of fitness of the various models proposed (see Table 5). The optimal model is selected based on the highest R-squared value and minimum AIC and BIC. Thus, the optimal model is the second one, which takes into consideration the interaction among variables of publicness and time effects.

**Fig. 1:**
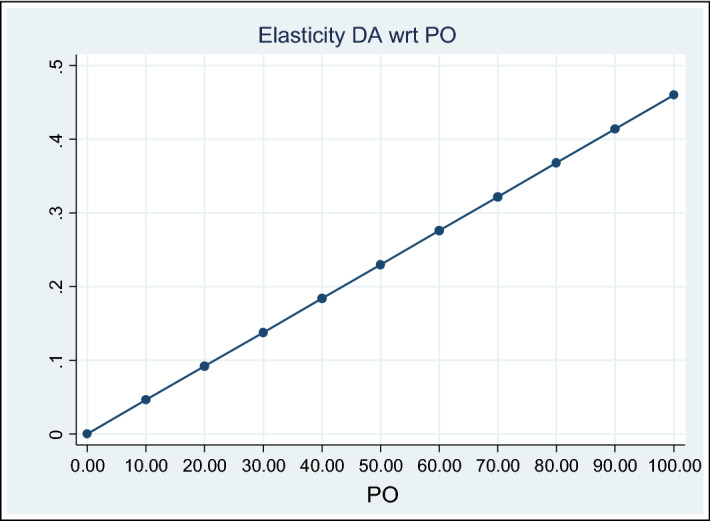
PO Elasticity of DA

Finally, the elasticity analysis^10^ was performed. Figure 1 below shows that EM is increasingly elastic to PO changes. Though the elasticity value is lower than one, the closer PO is to 100 per cent, the more EM is elastic with regard to that change. Consistent with property rights theory, public administrations have increasing relevance when public ownership increases. At the same time, this implies that the higher the level of public ownership, the more room SOE managers have to practise EM.

## Discussion

With the aim of investigating the relationship between publicness and EM in SOEs, this research involved the execution of statistical analysis on a sample of 1,200 Italian SOEs. The ‘conditional revenue model’ (Stubben, 2010) was used to estimate EM in the sample (Eq. 1). Then, the log of the absolute value of residuals (|DA|) from the Stubben model was regressed against three dimensions of publicness (ownership, control, and goal ambiguity), which were operationalised in four variables: public ownership (PO), administrative control (ADCONTR), financial control (FICONTR), and goal ambiguity (GA) (Eq. 2). Two issues had to be solved in the analyses. First, to overcome the problem of the unavailability of complete data on SOEs’ mission statements—used to measure mission comprehension, and thus to operationalise GA—Eq. (2) was run both including and excluding GA. As GA was not found to predict |DA|, it was ultimately excluded from the analysis. Both public administration and management control literature suggest that the multiplicity and vagueness of goals—deemed to be typical of public realms—would increase managers’ discretion to practise EM (Merchant & Van der Stede, 2012; Rainey & Jung, 2015). Nevertheless, empirical results highlight that the quality of financial accountability in SOEs is not influenced by the quality and quantity of goals settled at the strategic level. Therefore, a decoupling seems to emerge between strategic management and financial accountability in SOEs. On the one hand, this may be explained by the separation of positions and roles between top and financial managers in SOEs. Whereas the former set multiple and contrasting goals to pursue SOEs’ missions, the latter manipulate accounting information regardless of the leeway for discretion that higher goal ambiguity so determined. This also means that the behaviour of SOEs’ financial managers towards EM is explained by reasons other than goal ambiguity, as discussed later in this section. Second, the problem of multicollinearity between ADCONTR and FICONTR—two variables of the same dimension of publicness—was solved by dropping ADCONTR from the model because of its statistical irrelevance in explaining EM in SOEs.

Results show a statistically significant relationship between EM in SOEs and two out of four publicness variables analysed. Specifically, PO and FICONTR are causally linked to |DA|. As for PO, opposing arguments and contrasting empirical results from the extant literature led to the formulation of two different hypotheses. The results refute H1b, according to which the more numerous and heterogenous ‘eyes’ on SOEs, as well as the political rationales underpinning the appointment of SOEs’ managers and the weaker tax minimisation incentives, would have disincentivised SOEs’ managers to practise EM. On the contrary, results show that increasing levels of public ownership provide greater leeway for SOE managers to manipulate financial information, supporting H1a. These findings confirm that the increased number of SOE reporting addressees (Bruton et al., 2015; Grossi & Thomasson, 2015), along with relatively lower monitoring expectations and technical expertise (Jones, 1991; Koh, 2003), creates incentives for EM in SOEs. Additionally, this is consistent with property rights expectations (Asher et al., 2005), according to which residual public owners have fewer property rights and can therefore exert less pressure on SOE managers’ behaviours. Another aspect that could justify the positive relationship between EM and ownership is the ownership structure (Kazemian & Sanusi, 2015). In many cases, ownership is not concentrated in the hands of one or a few public administrations. This implies that managers have more power relative to owners, and more leeway to pursue their personal interests (Guo & Ma, 2015). The findings of this paper are consistent with those of Capalbo et al. (2014), Aharony et al. (2010) and Wang and Yung (2011), who suggest that SOEs manage their earnings similarly to or even more than their private counterparts do. Unlike these authors’, our results offer a possible interpretation of how the political authority exerted by public administrations through their shareholder status affects EM practices in SOEs. In particular, the level of public ownership is directly related to the possibility that SOEs’ managers will pursue their own self-interests through EM. This implies that political authority exerted through varying degrees of public ownership does not safeguard the multiplicity of public performance in SOEs. SOEs’ managers attribute relevance to and focus on financial-related performance more than other dimensions of performance that would increase the quality of SOEs’ financial accountability, such as transparency and fairness. Additionally, the result on the relationship between PO and EM in SOEs brings to the surface a doubt about the capacity/will of public administrations owning SOEs to exert political authority and pursue the public interest.

The second dimension of publicness studied here is control, i.e., the degree to which governmental policies and regulations influence organisations’ activities (Andrews et al., 2011; Bozeman, 2013; Moulton, 2009). As control can be exerted over both administrative and financial issues, two different aspects—and measures—of control were investigated. Building on insights from the publicness literature (Berry, 2005; Nutt & Backoff, 1993), it was hypothesised that higher degrees of political control over SOEs’ administrative issues would curb managerial discretion to adopt EM. This dimension was operationalised by making a distinction between organisations providing public essential services (entailing the existence of a greater administrative control) and organisations not providing that kind of services (less administrative control). Yet, results do not show any statistically significant relationship between ADCONTR and |DA|, leading to the rejection of H2. Therefore, the result suggests that control over SOEs’ activities does not affect managerial discretion in practising EM.

Political control is maintained in SOEs by virtue of the public relevance of their outputs, and apart from its statistical significance, the more it is exerted through rules and regulations on administrative aspects, the more SOE managers adopt EM. In other words, the awareness that their activities are being controlled by a multiplicity of sources induces SOE managers to manipulate financial information. This finding contradicts extant literature on EM in SOEs, according to which decreasing levels of monitoring compliance with formal rules and administrative regulations should provide greater leeway for EM (Aharony et al., 2010). On the contrary, any financial effect resulting from complying with those rules and regulations may be mitigated by the practice of EM (Healy & Wahlen, 1999; Königsgruber & Windisch, 2014).

The regulatory capture concept (Dal Bó, 2006; Laffont & Tirole, 1993) underpinned the hypothesis that higher degrees of control over financial issues would incentivise SOE managers to exploit accounting discretion to maximise their interests. Control over financial issues was operationalised by way of price regulation, assuming that SOEs operating in price-regulated industries are subject to higher degrees of such control. Results from regression analyses highlight the presence of a statistically significant and positive relationship between FICONTR and |DA|, leading to accept of H2b. These findings confirm expectations from the accounting literature, according to which price regulations are costly for firms and impact their accounting choices (Watts & Zimmerman, 1978). SOEs operating in price-regulated sectors are incentivised to exploit the information asymmetry and the related accounting discretion to increase the likelihood of approval of price increase requests (Bowman & Navissi, 2003; Lim & Matolcsy, 1999). This implies that control over financial issues affects managers’ decisions on accounting and reporting in SOEs more than control on activities, nevertheless with negative impacts on their financial accountability.

In summary, political authority exerted through control over administrative and financial issues is likely to undermine the financial accountability of SOEs. SOES’ managers perceive political authority exerted through administrative and financial controls as a worsening factor for SOEs’ financial performance. They consider potentially undesired financial results as not counterbalanced by the non-financial performance reached through reducing or eliminating EM, i.e., transparency and fairness.

In further investigations on the interaction among publicness variables included in the model performed, the interaction between PO and FICONTR is not statistically significant. Though PO and FICONTR alone explain EM in SOEs, their combination does not affect the quality of SOEs’ financial accountability. This implies that public ownership and price regulation affect EM but there is not an additional effect coming from their interaction.

In summary, SOEs’ publicness is either irrelevant or detrimental to the quality of SOEs’ financial accountability, depending on the dimension of publicness considered. Political authority is exerted on SOEs through varying degrees of manifestation of any of the analysed publicness dimensions maintained in these organisations with the aim of safeguarding the public interest in their activities and performance. Nonetheless, those publicness dimensions are at best irrelevant in explaining a managerial behaviour—EM—that affects the quality of SOEs’ financial accountability. Public ownership and political control over financial issues have detrimental effects on the quality of SOEs’ financial accountability. In other words, though SOEs are subjected to varying degrees of political authority to preserve the public interest, its deployment leads to opposite results, considering the key role of financial accounting and accountability in supporting governments decision-making (Bracci et al., 2015). Moreover, despite the public administration literature suggesting that publicness dimensions are likely to affect organisations’ behaviour also through their interaction (Goldstein & Naor, 2005; Nutt & Backoff, 1993), the results of this paper highlight that the combination of different dimensions through which political authority is exerted in SOEs does not affect the quality of their financial accountability.

In the end, the results of this paper raise a relevant question about political authority and accounting practices, i.e., EM. Is publicness able to influence managers’ behaviour in a way that ensures the quality of financial accountability in SOEs? The existence of a higher degree of publicness through the appropriate exerting of political authority proves to be a complex objective to pursue and reach. It is made of various dimensions that need to be governed to avoid unexpected effects. Exerting appropriately political authority depends on (1) how publicness dimensions are translated in practice by the subjects holding that authority, (2) possible contradictory effects deriving from each dimension, and (3) the complementarity among publicness dimensions.

## Conclusions

SOEs are privately run entities subject to some level of public control to safeguard the public interest in their activities (Asquer, 2014; Bel & Gradus, 2018; De Magalhães, 2010). Financial accountability is a major issue in SOEs, considering the amount of public resources that are invested in their activities, as well as the social implications of their performance (Royo et al., 2019; Shaoul et al., 2012). As accounting is the mechanism that allows accountability for finance, financial statements are the means through which SOEs’ financial accountability is fulfilled (Behn, 2001). In this regard, EM is a managerial intervention on accounting data that, exploiting the inherent degrees of discretion in accounting and reporting, undermines the quality of SOEs’ financial accountability.

The literature on EM in SOEs is relatively recent and has produced contrasting results (Capalbo et al., 2018). This paper addresses the need to increase understanding of the determinants of SOEs’ financial accountability, specifically focusing on the role played by the exercise of political authority in this respect. The findings highlight that the political authority to which SOEs are subject—through varying degrees of specific dimensions of publicness—is either detrimental or irrelevant to the quality of SOEs’ financial accountability.

This paper makes both theoretical and practical contributions. With reference to the former, this paper provides contributions that target specific kinds of literature and enhance interdisciplinary debate at the same time. On the one hand, it sheds light on accountability issues related to SOEs (Grossi et al., 2015) by providing an understanding of EM determinants that affect the quality of their financial accountability. As extant literature on EM in SOEs has primarily focused on either ownership structures (Capalbo et al., 2018) or country-specific features and exceptional circumstances (Lei & Wang, 2019; Wang & Yung, 2011), this paper broadens the scope of studies on EM explanations in SOEs, thus offering a valuable contribution to the accounting literature. Like other papers (Capalbo et al., 2014), this paper proposes discretionary accruals as a measure of the quality of SOEs’ financial accountability, but, differently from others, it also identifies a further perspective from which to study its determinants, i.e., publicness. Furthermore, conducting the research in a European country—in addition to being relevant for the purpose of this study—also enabled the study of EM in an empirical context not yet explored, thus contributing to overcoming the generalisability issues concerning previous research on this topic. On the other hand, this paper also adds to the publicness literature by proposing an adaptation of the dimensional publicness framework (Bozeman, 1987) and its measures to the SOE context. Furthermore, it highlights that accounting and reporting behaviour in SOEs is not affected by the interaction of different publicness dimensions, as the public administration literature would suggest (Boyne, 2002; Chun & Rainey, 2005; Goldstein & Naor, 2005). Additionally, publicness has not been conceived as a concept for analysing an organisation as a whole. Publicness and its dimensions could affect various organisations’ activities independently and differently. No less importantly, this paper melds two streams of literature in answering its research question, i.e., EM in SOEs and publicness. By doing so, the paper offers a timely response to the increasing calls to investigate accounting phenomena by adopting an interdisciplinary perspective (Jacobs, 2016). The combination of the literature of accounting and public administration brings publicness back into accounting research, and thus enhances the role of publicness as a conceptual space to investigate accounting phenomena. In particular, this paper promotes an understanding of EM in SOEs as an accounting phenomenon that occurs in an abstract arena where the public interest is pursued and where varying degrees of political authority may be exerted to safeguard it.

From a practical point of view, this paper proposes reflections on the role of political authority and how its influence can be exerted to improve financial accountability in SOEs. SOEs’ financial accountability is a matter of public relevance, with considerable implications regarding how SOEs’ financial performance is made accountable to both public administrations and citizens (Allini et al., 2016; Grossi & Thomasson, 2015; Royo et al., 2019; Shaoul et al., 2012). Since managers manipulate financial information, and as long as publicness dimensions affect such accounting behaviour, attention should be paid to their degree of presence in SOEs. In particular, the results of this paper suggest that efforts should be made to define policies and governance solutions able to contrast the detrimental impact of political authority on SOEs’ financial accountability. First, this paper delivers recommendations about the need to strengthen the monitoring power of SOEs’ residual owners, to contrast the detrimental impact of weak and fragmented ownership on SOEs’ financial accountability. In this regard, the requirement of financial and non-financial disclosures on SOEs’ websites may enhance the possibility for residual owners to monitor SOEs’ performances (Allini et al., 2016; Royo et al., 2019). Moreover, to the extent political authority is exerted through control over financial issues, this paper suggests that the financial accountability of SOEs operating in price-regulated sectors may be preserved by concurrently reducing the leeway for managerial discretion in accounting and reporting behaviours. Finally, considering the results from the interaction of publicness dimensions, solutions should be found in terms of governance arrangements able to incentivise the construction of an environment in which the detrimental effect of the different publicness dimensions on EM is mitigated from their combination.

On closer inspection, the publicness perspective adopted in this paper allows for shifting the focus from the effect of contextual variables on EM in SOEs to the effect that such accounting phenomenon is likely to have in those arenas where the public interest is pursued. In times at which governments assess the suitability of investing in/disinvesting from SOEs also on the grounds of their financial performance (Bracci et al., 2015; Lapsley et al., 2015), this paper claims that it is relevant to understand whether and why governmental decision-making in that respect may be biased by the active interventions of managers in the process of financial performance representation. In turn, such possible biases hinder the democratic accountability relationship between governments and citizens (Grossi et al., 2015). This suggestion is particularly relevant during and after the Covid-19 pandemic, as there is a growing call for an increased role of public administrations in the economy, especially through the ownership of organisations that are considered relevant for social and economic recovery. As such, by exploring the implications of accounting behaviours on policymakers, politicians’ decision-making, and on the democratic values and accountability relationships, this paper also represents an attempt to emancipate public sector accounting scholarship (Bruns et al., 2020; Steccolini, 2019).

It should be stressed that the results of the analysis reported in this paper could be affected by the operationalisation of publicness dimensions. Future research may replicate this study by using additional and different measures of the same publicness dimensions. Moreover, the empirical setting for conducting this study is the Italian context, a relevant one for the purpose of this study, and the sample included in the analysis is relatively broad for a single country. Nonetheless, the generalisability of its findings may be broadened by replicating this study in other institutional settings. Despite the relevance of the context chosen and the exploratory purpose of this paper justifying this methodological choice, it would be appropriate to replicate this study in an empirical context for which funding—as a dimension of publicness—can be measured according to its original conception.

Notably, while this paper has answered its core research question, its findings shed light on the need to further investigate the role of publicness in explaining the quality of SOEs’ financial accountability. Therefore, future studies are encouraged to embark on such a research path by adopting interpretative and inductive approaches to investigate the reasons behind the relationships between each publicness dimension and EM adoption in SOEs as brought to light in this paper. This promises to deliver relevant conceptual and empirical insights on the impact of political authority on accounting and reporting behaviour.

## Appendix

Tables 6, 7, 8, 9, 10.

**Table 6:** Classification of economic sectors (Ateco 2007) in regulated and essential

CODE	DESCRIPTION	REGULATED SECTORS	ESSENTIAL SERVICES
D	Supply of electricity, gas, steam and air conditioning		
35	Supply of electricity, gas, steam, and air conditioning	x	x
E	Supply of water; sewer networks, waste management and remediation activities		
36	Water collection, treatment, and supply	x	x
37	Management of sewer networks	x	x
38	Waste collection, treatment, and disposal activities; recovery of materials	x	x
39	Remediation activities and other waste management services	x	x
F	Buildings		
43	Specialized construction work		x
H	Transport and storage		
49	Land transport and transport via pipelines	x	x
50	Sea and water transport	x	x
51	Airplane transport	x	x
52	Warehousing and transport support activities	x	x
53	Postal services and courier business	x	x
J	Information and communication services		
58	Editorial activities	x	x
59	Production of films, videos and television programs, music, and sound recordings	x	
60	Programming and broadcasting activities	x	x
61	Telecommunications	x	x
K	Financial services and insurance		
64	Financial services activities (excluding insurance and pension funds)	x	
65	Insurance, reinsurance and pension funds (excluding compulsory social insurance)	x	
66	Activities auxiliary to financial services and insurance activities	x	
M	Professional, scientific, and technical activities		
70	Business management and management consulting activities		x
N	Rental, travel agencies, business support services		
82	Support activities for office functions and other business support services		x
O	Public administration and defense; compulsory social insurance		
84	Public administration and defense; compulsory social insurance		x
85	Education		x
Q	Health and social assistance		
86	Health care		x
87	Residential social work services		x
88	Non-residential social assistance		x
R	Artistic, sports, entertainment and fun activities		
91	Libraries, archives, museums, and other cultural activities		x
S	Other service activities		
96	Other services for the person		
96.03	Funeral home services and related activities		x
96.04	Spa		x

**Table 7:** Descriptive statistics per year for the whole sample

Year	Stats	Variables					
		PO	GA	SIZE	ROA	LEV	NOI
2010	Mean	85.10	27.36	3.72207	3.083833	8.814592	− 325.784
	p50	99.90667	26.30	3.656969	2.165	3.785	− 50.594
	sd	21.66136	6.508452	.9320304	10.08178	22.55315	16,650.38
	Min	17.72	11.47	1.003245	− 87.3	− 129.11	− 492,720
	Max	100	67.9	7.718759	91.13	324.08	262,104
2011	Mean	85.10123	27.35706	3.748183	3.263583	9.086742	− 1175.33
	p50	99.90667	26.3	3.698294	2.36	3.68	− 56.26
	sd	21.66136	6.508452	.9275843	9.876853	28.69562	25,363.45
	Min	17.72	11.47	1.117371	− 106.84	− 243.4	− 697,404
	Max	100	67.9	7.724321	71.9	401.97	219,305
2012	Mean	85.10123	27.35706	3.758694	3.181283	10.01726	− 265.901
	p50	99.90667	26.3	3.69515	1.9	3.695	− 34.627
	sd	21.66136	6.508452	.9200376	8.579059	60.44215	14,601.7
	Min	17.72	11.47	1.632427	− 68.79	− 951.46	− 409,525
	Max	100	67.9	7.787174	100.75	1482.17	219,139
2013	Mean	85.10123	27.35706	3.7617	3.2118	10.04168	− 241.343
	p50	99.90667	26.3	3.672019	2.225	3.445	− 43.5815
	sd	21.66136	6.508452	.9135128	11.84207	66.75562	9119.177
	Min	17.72	11.47	1.764512	− 232.77	− 304.82	− 161,156
	Max	100	67.9	7.798584	72.19	2107.56	144,536
2014	Mean	85.10123	27.35706	3.76336	3.4545	7.82895	238.9749
	p50	99.90667	26.3	3.682323	2.21	3.38	− 41.7935
	sd	21.66136	6.508452	.9150621	10.73408	35.52718	11,586.86
	Min	17.72	11.47	1.669605	− 141.05	− 773.3	− 36,035.8
	Max	100	67.9	7.828724	76.21	464.67	305,033
2015	Mean	84.78917	27.35706	3.763963	3.113525	6.458367	420.3283
	p50	99.98	26.3	3.673321	2.305	3.25	− 42.7665
	sd	22.29955	6.508452	.9121626	22.94728	31.97049	16,329.32
	Min	.96	11.47	1.777572	− 732.1	− 806.73	− 39,439
	Max	100	67.9	7.844612	64.75	304.86	480,991
2016	Mean	85.78307	27.35706	3.767314	3.570367	11.95149	1367.225
	p50	100	26.3	3.663159	2.34	3.1	− 45.4325
	sd	21.82321	6.508452	.9085899	9.606124	155.6453	32,172.88
	Min	.72	11.47	1.226445	− 125.44	− 461.47	− 37,478
	Max	100	67.9	7.868818	61.05	5304.61	783,921
2017	Mean	84.73145	27.35706	3.771403	3.450242	5.945817	1342.126
	p50	100	26.3	3.676877	2.18	2.975	− 38.7135
	sd	23.42828	6.508452	.9090363	14.45717	32.43142	30,204.15
	Min	0	11.47	1.232437	− 389.88	− 672.9	− 41,974
	Max	100	67.9	7.879718	67.28	466.21	943,623

**Table 8:** Robust regression with both variables of control dimension

VARIABLES	Robust regression
	|DA|
PO	0.00484***
	(0.00082)
ADCONTR	0.08954
	(0.09271)
FICONTR	0.31695***
	(0.09401)
SIZE	− 0.5199402***
	(0.02236)
ROA	0.00213
	(0.00131)
LEV	0.0000808***
	(0.0004)
NOI	1.75e-06
	(1.39e-06)
Constant	− 4.42409***
	(0.10804)
Observations	9,599
R-squared	0.0703
Fstat	89.35
Prob > F	0.0000

Robust standard errors in parentheses

***p < 0.01; **p < 0.05; *p < 0.1

**Table 9:** Breusch-Pagan/Cook-Weisberg test for heteroskedasticity

Variables	Chi2	df	p
PO	168.28	1	0.0000
ADCONTR	311.99	1	0.0000
FICONTR	390.41	1	0.0000
SIZE	4084.83	1	0.0000
ROA	1716.93	1	0.0000
LEV	14.20	1	0.0002
NOI	31.62	1	0.0000
Simultaneous	7014.56	7	0.0000

Ho: Constant variance

**Table 10:** Regression with and without GA

Variables	With GA	Without GA
PO	0.0038***	0.0038***
FICONTR	0.4722***	0.4725***
GA	− 0.0015	
SIZE	− 0.3746***	− 0.3772***
ROA	0.0072*	0.0071*
LEV	0.0004	0.0004
NOI	0.0000***	0.0000***
_cons	− 4.7229***	− 4.7544***
F	27.9861	32.6627

*p < .1; **p < .05; ***p < .01

## Footnotes

1 SOEs providing no-market services (e.g., cultural services) may be allowed to receive direct endowments from governments in many jurisdictions. Though this would make it relevant to include the funding variable in the theoretical framework, it was also considered that its measurement in the SOE context would be unfeasible.

2 The sample comprises limited companies, as this typology represents about the 74% of all Italian SOEs. In addition, about 92% of the employees of SOEs are hired in limited companies (ISTAT 2017).

3 According to Italian Law n. 146/1900 (art. 1), essential services are ‘those aimed at guaranteeing constitutionally protected rights to life, health, freedom and safety, freedom of movement, assistance and social security, education and freedom of communication’. Due to the social relevance of essential services, public authorities exert greater control over their activities.

4 Beginning 1 January 2008, the Italian National Institute of Statistics (ISTAT) adopted this economic activity classification, the Italian version of the European nomenclature expressed in Nace Rev.2 (CE Regulation n. 1893/2006). Accordingly, companies can be classified based on the sectors they pertain to, as reflected in the ATECO coding.

5 Law 14 November 1995, n. 481, on competition and regulation of public utility services.

6 Pursuant to legislative decree no.33/2013.

7 The values of residuals resulting from Eq. (1) were not normally distributed. In order to make these values closer to a normal distribution, their logarithms were considered. Absolute values are used, because EM is here considered as any alteration – positive or negative – to the financial performance representation.

8 Though data were extracted from 2009 to 2017, the first year served as a basis for estimating annual variations of variables.

9 The alternative strategy would have been the imputation of missing data (Raghunathan et al., 2001), but this does not seem appropriate given the specificity of mission statements to a given organization (Mullane, 2002).

10 The elasticity of |DA| was computed based on the semi-elasticity of its value given that these values are the log of the absolute values of the errors from Eq. (1).
